# A study of soluble protein and sulfhydryl levels in the rat liver during rapid normal and premalignant growth.

**DOI:** 10.1038/bjc.1968.41

**Published:** 1968-06

**Authors:** M. F. Argus, J. A. Walder, J. A. Fabian, J. C. Arcos


### A STUDY OF SOLUBLE PROTEIN AND SULFHYDRYL LEVELS IN THE RAT LIVER DURING RAPID NORMAL AND PRE-MALIGNANT GROWTH

MARY F. ARGUS, JANIS A. WALDER, JUDITH A. FABIAN, AND J. C. ARCOS

*From the Seamen's Memorial Research Laboratory, U.S. Public Health Service Hospital, 210 State Street, New Orleans, and the Department of Medicine (Biochemistry), Tulane University School of Medicine, New Orleans, Louisiana, U.S.A.*

Received for publication February 3, 1968

FOLLOWING the demonstration by Miller and Miller (1947) and Miller, Miller, Sapp and Weber (1949) that carcinogenic azo dyes are bound covalently to rat liver proteins before the appearance of tumors, the cytoplasmic *h* protein components of this combination (Sorof and Cohen, 1951; Wirtz and Arcos, 1958) have been extensively investigated. During liver carcinogenesis by the aminoazo dyes and by 2-acetylaminofluorene, the $h_2$ liver proteins contain the greatest portion of the soluble carcinogen-protein conjugates (Sorof, Young and Ott, 1958; Sorof, Young and Fetterman, 1960), and there is at the time of maximum dye binding an increase in the total *h* protein fraction of the liver (Sorof, Young and Ott, 1958). In contrast, the primary tumors induced by these carcinogens do not form $h_2$ protein-carcinogen conjugates (Sorof, Young McCue and Fetterman, 1963; Sorof, Young, McBride and Coffey, 1965), and the *h* fraction is considerably decreased in these hepatomas (Sorof and Cohen, 1951; Sorof, Young and Ott, 1958).

It has been hypothesized that carcinogens combine with specific growth-controlling proteins, the deletion of which gives rise to malignant cells (Miller and Miller, 1953; Potter, 1958), and certain $h_2$ proteins of rat liver were postulated to be involved in regulation of cell multiplication (Bakay and Sorof, 1964). Freed and Sorof (1966) provided evidence that the $h_2$ liver proteins do function in normal cells as metabolic regulators: isolated $h_2$ protein fraction strongly inhibits the growth of L strain mouse fibroblasts in suspension tissue culture, and the inhibition of cell multiplication is reversed by removal of the $h_2$ proteins. The inhibitory fraction centred at the slow $h_2$ proteins has been recently identified as arginase (Sorof, Young, Luongo, Kish and Freed, 1967).

The present investigations attempt to gain specific information on the possible role of the *h* proteins in the rapid normal growth of regenerating liver, in the rapid liver growth induced by polycyclic aromatic hydrocarbons, and in nitrosamine hepatocarcinogenesis. Thus, a study of the electrophoretic patterns of soluble rat liver proteins at various time intervals was carried out following partial hepatectomy and following intraperitoneal injection of a single dose of 20-methylcholanthrene. Electrophoretic patterns were also studied in soluble liver proteins obtained from rats which were continuously administered the hepatic carcinogen, diethylnitrosamine. The sulfhydryl levels in the soluble protein preparations and the mitotic indexes of the liver tissues were measured in order to correlate the electrophoretic patterns with these parameters of mitosis.

### MATERIALS AND METHODS

### *Care of animals, partial hepatectomy, and administration of compounds*

Sprague-Dawley male rats (Holtzman Co., Madison, Wis., USA) were maintained on Purina laboratory chow for the partial hepatectomy (weight range 230–260 g.) and diethylnitrosamine experiments (initial weight range 180–250 g.), and on a semi-synthetic diet (Arcos, Gosch and Zickafoose, 1961) for the experiments with 20-methylcholanthrene (weight range 40–60 g.). For both diets and all animals, food and water were given *ad libitum.*

The partial hepatectomies, in which two-thirds of the livers were removed, were performed under light ether anesthesia. Sham operations (for controls) were performed by opening the animal, massaging the liver, and suturing the incision, using the same operative procedures as for the partial hepatectomies.

20-Methylcholanthrene (Eastman No. 4383) was administered as freshly prepared solutions in corn oil (2 mg. hydrocarbon per ml.) by intraperitoneal injection at the level of 1 mg. per 50 g. body weight. Diethylnitrosamine (Eastman No. 7341) was administered in the drinking water, each animal consuming an average of 650 $\mu$g. carcinogen per day.

### *Preparation and standardization of soluble liver protein lyophilizates*

The rats were killed 6, 24, 37, 48 and 96 hours following partial hepatectomy; 6, 12, 24, 48, and 96 hours following 20-methylcholanthrene injection; and following 1, 2, 3, 4, 6, 8, and 10 weeks of diethylnitrosamine administration. In addition to the aforementioned sham operated controls which were killed 38 hours postoperatively, 40–60 g. rats receiving corn oil alone intraperitoneally and killed at 48 hours served as controls for the 20-methylcholanthrene experiments, and 230–250 g. rats served as untreated controls for the diethylnitrosamine studies.

Keeping constant the time of the day at which the partial hepatectomies were performed, the time of killing occurred during daylight hours for all of the time intervals studied except 37 hours. Since mitotic activity is generally lower during the night than during the day, it was considered possible that this variation in mitotic activity could influence the parameters being studied. Thus, one-half of the 37 hour-interval animals were partially hepatectomized at night so that they could be killed during daylight hours.

The rats were weighed, and killed by severing the spinal column following a blow on the head. The liver of each rat was perfused *in situ* with ice cold physiological saline, then rapidly removed, weighed, reweighed after a small piece was taken for histology, and the remainder of the liver was placed in ice cold 0·25 M sucrose for homogenization.

From the liver tissues, 40 % homogenates were prepared in 0·25 M sucrose using an Elvejhem-Potter homogenizer at 3° C. The homogenates were centrifuged at 105,000 *g* for 60 minutes in a Spinco L preparatory ultracentrifuge; the supernatant fractions (approximately 5–10 ml.) were decanted into 250 ml. round bottom flasks, quick frozen in acetone (previously cooled to —70° C. with dry ice), and the frozen material was lyophilized in a Virtis Manifold type freeze-dryer. The effective protein content of each lyophilizate was determined following Lowry, Rosebrough, Farr and Randall (1951) using denatured total rat liver supernatant protein as standard.

Six separate lyophilizates were prepared for the untreated controls and for rats administered diethylnitrosamine for each of the time intervals. Four separate lyophilizates were prepared for animals at each of the time intervals following injection of 20-methylcholanthrene and for the controls receiving corn oil. The number of separate lyophilizates for the partial hepatectomy study varied from 6 to 11 (see column 2, Table I). A single animal was used for the preparation of each separate lyophilizate except in the case of the partially hepatectomized rats and of the weanling rats used in the 20-methylcholanthrene experiments, where in some cases the livers from 2 to 6 animals were pooled for the preparation of each separate lyophilizate because of the small amounts of liver tissue available.

*Electrophoresis*

The zone starch electrophoresis apparatus and the procedures for filling with starch slurry and equilibrating are identical to those used by Arcos and Arcos (1958).

After the initial equilibration for 4 hours, a 1 cm. long segment in the starch block was exposed by cutting the Parafilm cover at the 24th cm. from the negative pole, and the starch slurry was removed from this portion. A slurry, having the consistency of the block, prepared from the lyophilizate to be studied (which had been dissolved in 0·6–0·8 ml. of barbituric buffer) and starch, was used to fill up the trench. The amount of lyophilizate used for an electrophoresis was such as to contain a standard 30 mg. of protein. The segment containing the protein was covered with Parafilm, the case closed, and after equilibrating for 10 minutes the current was turned on. The conditions of electrophoresis were: 225 v and 11–13 mA per $1 \times 2 \times 31$ cm. block for 20 hours.

At the end of the run the block was cut into 1 cm. pieces, which were dropped into separate centrifuge tubes, each containing 2 ml. of ice-cold 0·013 M KCl and 0·002 M phosphate buffer, pH 7·0. The negative end was always tube No. 1. The starch pieces were broken up by stirring once, mechanically, with a glass paddle; the suspensions were centrifuged in the cold, and 0·10 ml. volumes from each supernatant fluid were assayed for protein content by the same colorimetric method used above (Lowry, Rosebrough, Farr and Randall, 1951). These experiments were carried out in duplicate per separate lyophilizate for the 20-methylcholanthrene and diethylnitrosamine studies and their controls, and in triplicate for the partial hepatectomies and sham operated controls.

Each individual electrophoresis was plotted on high-grade millimetric graph paper as absorbance *versus* length (in cm.) of the starch block (exemplified in Fig. 1). The curves were carefully cut out and weighed on an analytical balance. The weight of graph paper underlying the entire curve was equivalent, for each particular lyophilizate, to 30 mg. of protein which was the amount of protein inserted into the starch block. The amount of *h* protein fraction was then determined for each electrophoresis experiment by weighing the portion of graph paper falling under the *h* protein shoulder (Fig. 1), and referring this weight to that of the total curve by direct proportion.

*Determination of the sulfhydryl content of the lyophilizates*

The sulfhydryl content of the lyophilizates was determined following a modification (Argus, Arcos, Mathison and Alam, 1966) of the procedure of Hellerman,

FIG. 1—Typical electrophoretic pattern of soluble rat liver protein obtained in the present studies where lyophilizate containing a standard 30 mg. protein was electrophorized on starch block. No. 1 is the negative end.

Chinard and Dietz (1943) and Chinard and Hellerman (1954) by reacting the —SH groups of exactly 5 mg. protein with an excess of iodosobenzoic acid, and then back-titrating the remaining iodosobenzoic acid iodometrically. The concentration of —SH groups was expressed as mg. cysteine equivalent per mg. protein, calculated by direct proportion where 132 mg. iodosobenzoic acid is equivalent to 121·16 mg. cysteine.

*Mitotic index*

A small piece of liver (approximately 5 × 5 × 10 mm.) from each animal in the experiments was preserved in Mossman fixative (acetic acid-ethanol-formalin-water; 2 : 4 : 4 : 30 parts per volume). The tissue samples were dehydrated through one change each of 80% ethanol, 95% ethanol, acetone, and xylene, embedded in Paraplast embedding medium, and sectioned as 4 $\mu$. As a precaution against counting one mitotic figure twice, alternate sections from the ribbon were mounted, 5 sections per slide. Staining was with hematoxylin and eosin to bring out the nucleus. The total number of hepatic cell nuclei in the field delimited in a 10 $\times$ 10 mm. reticle under oil immersion (2000 $\times$ magnification), and the number of these hepatic cells in mitosis (prophase, metaphase or anaphase, and telophase) were counted for 3 randomly selected fields on each slide. Approximately 50 cells were counted in each of these fields. The mitotic index was expressed as the per cent of the total nuclei counted which were in mitosis. The mitotic indexes for 8 individual rats were averaged for the controls and for each time interval studied in the partial hepatectomy and 20-methylcholanthrene experiments; in the diethylnitrosamine studies values from 6 rats were used for each time interval.

### RESULTS

### *Partial hepatectomy*

Table I summarizes the effects of partial hepatectomy on the soluble rat liver proteins. Column 3 shows the change in the amount of *h* protein fraction expressed as milligram *h* protein times 100 per milligram total supernatant protein.

TABLE I.—*Effect of Partial Hepatectomy on Soluble Rat Liver Proteins and Hepatic Mitotic Index*

| Hours after partial hepatectomy | Number of lyophilizates | percentage *h* protein‡ | Probability§ | Mg. soluble Protein per g. lyophilizate | Mg. cysteine equivalent per mg. protein | Average liver weight per body weight ratio | Mitotic index‖ |
|---|---|---|---|---|---|---|---|
| 0* | 9 | 15·26 | — | 271 | 0·035 | — | 3·5 |
| 6 | 6 | 13·87 | $0·4>p>0·3$ | 298 | 0·028 | 0·013 | 10·6 |
| 24 | 11 | 9·74 | $0·01>p>0·001$ | 178 | 0·038 | 0·015 | 13·2 |
| 37 | 4 | 7·23 | $0·001>p$ | 206 | 0·044 (37 and 37†) | 0·020 (37 and 37†) | 11·0 |
| 37† | 4 | 7·64 | $0·001>p$ | 190 | | | 11·6 |
| 48 | 8 | 11·76 | $0·10>p>0·05$ | 176 | 0·047 | 0·023 | 14·0 |
| 96 | 9 | 13·81 | $0·3>p>0·2$ | 250 | 0·038 | 0·029 | 5·9 |

* Sham operated controls.
† Animals killed at 11 p.m.; all other animals were killed during daylight hours.
‡ Mg. *h* protein $\times$ $10^2$/mg. total supernatant protein.
§ Based on the null hypothesis for a true difference in per cent *h* protein between experimental (partially hepatectomized) and control groups' values.
‖ Each value is the average of 8 individual rats.

Compared to the control values, there is a gradual decrease in the percent of *h* protein up to 37 hours after partial hepatectomy, followed by a gradual increase approaching the control level by 96 hours. The decrease in *h* protein fraction has an unequivocal statistical significance at 24 and 37 hours (column 4).

Variations of the total supernatant protein per gram of lyophilizate are given in column 5 (Table I). Since the lyophilizates were prepared under standardized conditions, these variations represent actual changes in soluble *versus* particulate cell components in the liver tissue. There is a large decrease in the amount of soluble total protein at 24, 37 and 48 hours. In view of this it should be pointed out that the observed decreases in the *h* protein fraction represent true changes, since amounts of lyophilizates containing a standard 30 mg. of protein were used for each electrophoresis. Both, the decrease of *h* protein and the decrease of total soluble protein correspond to the time of highest mitotic activity as measured by the mitotic index (column 8).

Column 6 of Table I shows the change of the sulfhydryl level in the total soluble proteins of rat liver during regeneration, following partial hepatectomy. After a small initial decrease at 6 hours there is a gradual increase of the sulfhydryl level reaching a peak at 48 hours, followed by a decline at 96 hours. The probabilities calculated for the difference between 0 + 6 hours as one population and 37 or 48 hours is $0{\cdot}05 > p > 0{\cdot}02$, and for the difference between 48 and 96 hours $0{\cdot}10 > p > 0{\cdot}05$.

As indicated in column 7 there was a gradual increase with time (following partial hepatectomy) of the liver weight per body weight ratio, indicating a rapid and even rate of regrowth of the liver; by 96 hours the liver had regained approximately 68 % of its original weight.

At 37 hours following partial hepatectomy no significant differences were found for any of the parameters studied in the two groups killed during daylight and at night.

*Intraperitoneal injection of 20-methylcholanthrene*

Up to 96 hours following injection of 20-methylcholanthrene, no significant change was found in the *h* protein level of the soluble rat liver proteins (Table II, column 2). The probability for true difference between the controls and each of the time intervals studied is between either 0·20–0·10 or 0·30–0·20.

TABLE II.—*Effect of a Single Intraperitoneal Injection of 20-Methylcholanthrene on Soluble Rat Liver Proteins and Hepatic Mitotic Index*

| Hours following injection of 20-methyl-cholanthrene | Percentage *h* protein†‡ | Mg. soluble protein per g. lyophilizate† | Mg. cysteine equivalent per mg. protein† | Average liver weight per body weight ratio | Mitotic index§ |
|---|---|---|---|---|---|
| 0* | 16·91 | 257 | 0·024 | 0·050 | 4·9 |
| 6 | 14·93 | 260 | 0·041 | 0·044 | 6·5 |
| 12 | 14·44 | 220 | 0·045 | 0·045 | 10·9 |
| 24 | 17·74 | 187 | 0·038 | 0·054 | 13·1 |
| 48 | 14·33 | 202 | 0·033 | 0·053 | 10·5 |
| 96 | 16·62 | 212 | 0·035 | 0·064 | 7·8 |

* Controls injected with corn oil (used as solvent).
† Each value is the average obtained with 4 individual lyophilizates.
‡ Mg. *h* protein × $10^2$/mg. total soluble protein.
§ Each value is the average from 8 individual rats.

As with partial hepatectomy, 20-methylcholanthrene caused a substantial decrease in the protein content of the lyophilizate (Table II, column 3), the lowest value occurring at 24 hours. The time of decrease in total soluble protein paralleled the time of greatest mitotic activity (column 6). The only significant increase in the liver weight-body weight ratio occurred at 96 hours (column 5).

Just as during liver regeneration, the sulfhydryl level following 20-methylcholanthrene injection (Table II, column 4) varied roughly inversely with the protein content of the lyophilizates. The probability calculated for the true difference between the corn oil injected controls and the animals killed 12 hours after injection of 20-methylcholanthrene is $0{\cdot}01 > p > 0{\cdot}001$, and between the values at 12 hours and 48 + 96 hours as one population is $0{\cdot}02 > p > 0{\cdot}01$.

*Administration of diethylnitrosamine*

Columns 2 and 3 of Table III give the levels of *h* protein and the protein content of the lyophilizate of soluble rat liver protein during continuous administration of

TABLE III.—*Effect of Prolonged Oral Administration of Diethylnitrosamine on Soluble Rat Liver Proteins and Hepatic Mitotic Index*

| Weeks of diethylnitrosamine feeding | Percentage *h* protein*† | Mg. soluble protein per g. lyophilizate* | Mg. cysteine equivalent per mg. protein* | Mitotic index* |
|---|---|---|---|---|
| 0 | 17·12 | 240 | 0·026 | 5·4 |
| 1 | 35·58 | 339 | 0·027 | 5·7 |
| 2 | 26·75 | 308 | 0·032 | 6·1 |
| 3 | 23·99 | 330 | 0·025 | 6·2 |
| 4 | 25·48 | 312 | 0·029 | 6·8 |
| 6 | 20·30 | 307 | 0·032 | 7·8 |
| 8 | 21·58 | 283 | 0·033 | 9·9 |
| 10 | 20·81 | 315 | 0·033 | 11·3 |
| Tumor | 12·25 | 135 | — | — |

* Each value is the average from 6 individual rats.
† Mg. *h* protein $\times 10^2$/mg. total soluble protein.

diethylnitrosamine. The most dramatic change occurred when the animals had received the carcinogen for 1 week, at which time both parameters showed a steep rise. This was followed by a slow decrease. The statistical significance of the increase in the amount of *h* fraction at 1 week and of the subsequent decrease, leveling off at 6, 8 and 10 weeks is for both, between untreated controls and 1 week, and between 1 week and 6, 8, 10 weeks taken as one population, $p \cong 0{\cdot}001$. Both, the amount of *h* protein fraction and the total soluble protein were drastically reduced in hepatic tumors induced by diethylnitrosamine administration, as compared to normal control rat liver.

Change in the sulfhydryl level of the soluble cytoplasmic proteins is shown in Table III, column 4. There is first a rise between 0 and 2 weeks, then a dip at 3 weeks, followed again by a steady, non-reversible increase up to 10 weeks. All these changes are statistically significant: between 0 and 2 weeks, between 2 and 3 weeks, and between 3 and 8 weeks the probabilities calculated for true differences are all $0{\cdot}05 > p > 0{\cdot}02$.

Following a slight dip in the mitotic index at 1 week (coinciding with the dramatic rise in the amount of *h* protein and increase of total soluble proteins), the mitotic activity of the hepatic cells increased steadily through the 10 weeks of diethylnitrosamine administration (Table III, column 5). At 10 weeks the mitotic index was more than twice that after 1 week of carcinogen feeding. The liver weight-body weight ratio of the animals receiving diethylnitrosamine slowly but steadily decreased. The rate of decrease, however, was not significantly different from that of normal growing rats (Setnikar and Magistretti, 1965).

### DISCUSSION

*Partial hepatectomy*

The marked increase in mitotic index following partial hepatectomy, with peaks at 24 and 48 hours, is in agreement with the results of Becker and Broome (1967) who reported mitotic waves 30 and 50 hours after partial hepatectomy, and with the findings of Canellakis, Jaffe, Mantsavinos and Krakow (1959) who indicated the existence of mitotic peaks at 24, 48 and 72 hours. The latter authors suggested that these mitotic peaks may not be due to diurnal variations, but reflect periodicity of mitosis inherent to rapid liver regeneration proper. Failure in the present study to find a difference in the liver mitotic index for hepatectomized animals killed (37 hours postoperatively) during daylight hours and during the night indicates that the diurnal increase in mitotic index of normal liver is here masked by the increased mitotic activity of rapidly regenerating liver.

The drastic decrease in the per cent *h* protein which parallels the increased mitotic activity is in contrast with the results of Sorof, Claus and Cohen (1951) who reported no change in the *h* fraction after partial hepatectomy in studies conducted at various time intervals for 8 days. The decrease of *h* protein found in the present experiments does, however, coincide with the increased rate of DNA synthesis reported by Hecht and Potter (1956); a maximum rate of DNA synthesis was observed by them in rat liver 24–36 hours following partial hepatectomy.

The correlation between the time of greatest decrease of the *h* protein fraction and the time of highest rate of nucleic acid synthesis (Hecht and Potter, 1956) substantiates the possibility that the *h* protein, or a fraction thereof, may be a regulatory factor in protein synthesis, which process is requisite for cellular hypertrophy. If the *h* protein fraction is an inhibitor of nucleic acid (and thus protein) synthesis, then absence or reduced amounts of the *h* fraction should allow protein synthesis to continue at an accelerated rate leading finally to hypertrophy of the cells. Decrease of the nucleo-cytoplasmic ratio would then trigger the mitotic mechanism (Hämmerling, 1939, 1953). The nucleo-cytoplasmic ratio corresponding to resting cells would be thereby temporarily restored. The cyclic processes, hypertrophy and mitosis, would continue as long as inhibitors of the former are maintained at a low level, until the original liver weight-body weight ratio is reestablished. Furthermore, since low levels of *h* fraction parallel low levels of total soluble cytoplasmic proteins (Table I, column 5) it is likely that the decrease of the nucleo-cytoplasmic ratio is not so much due to the production of all cytoplasmic components, but rather that the synthesis of particulate high-speed sedimentable components (endoplasmic reticulum and mitochondria) predominates over the soluble ones. This is actually not unexpected in view of the fact that cytoplasmic protein synthesis occurs in the endoplasmic reticulum and that the process requires ATP which is produced in the mitochondria.

The increase noted in the sulfhydryl level of soluble liver proteins following partial hepatectomy is in agreement with the data of Hopsu and Harkonen (1960) who showed by histochemical methods that there is an overall increase in the actual number of sulfhydryl groups in total liver protein during regeneration, and with the findings of Fraser and Cater (1967) that the level of acid-soluble —SH during liver regeneration in the rat increases above normal by 12 hours after hepatectomy and remains elevated until after the first wave of mitosis at 28–30 hours.

In the present studies the period of highest —SH level (37–48 hours) was found to overlap with the time of lowest soluble cytoplasmic protein content and minimum *h* protein level. It also correlates with the time of the highest level of carbamyl phosphate-aspartate transcarbamylase activity as reported by Calva and Cohen (1959). Carbamyl aspartate is an intermediate in a series of reactions leading to the biological synthesis of pyrimidine nucleotides in the production of RNA and DNA. That the 48 hour time interval is indeed the time of the highest rate of pyrimidine nucleotide synthesis is also indicated by the observation that the rate of catabolism of exogenously added uracil is at the lowest at this time interval following partial hepatectomy (Canellakis, 1957). The same was found with another pyrimidine component, thymine (Canellakis, Jaffe, Mantsavinos and Krakow, 1959).

The increase in the level of carbamyl asparate found by Calva and Cohen (1959) is in agreement with the speculation of Sorof, Young, Luongo, Kish and Freed (1967) concerning the manner in which a decrease in the level of the inhibitory fraction of *h* protein (or arginase) could help to bring about tissue proliferation. As a result of decreased arginase activity not only would more arginine be available for the synthesis of cell proteins, but less ornithine would be produced and thus less carbamyl phosphate would enter the urea cycle. More carbamyl phosphate would, therefore, be available to the alternate pathway (through carbamyl aspartate) for the synthesis of pyrimidine nucleotides and production of nucleic acids.

*Intraperitoneal injection of* 20-*methylcholanthrene*

Unlike the normal rapid liver growth following partial hepatectomy, induced rapid liver growth resulting from an intraperitoneal injection of 20-methylcholanthrene is not accompanied by any significant change in the level of the *h* protein fraction (Table II, column 2). As with partial hepatectomy, 20-methylcholanthrene does cause, however, a drastic decrease in the protein content of the lyophilizates (Table II, column 3). Therefore, the increasing liver weight-body weight ratio observed between 24 and 96 hours, compared to the control value (Table II, column 5), is probably predominently due to increased synthesis of sedimentable cell components. Consistent with this are the observations (e.g., Arcos, Conney and Buu-Hoi, 1961) that following intraperitoneal injection of 20-methylcholanthrene or certain other polycyclic hydrocarbons there is a considerable increase in the synthesis of liver microsomal drug metabolizing enzymes and of total liver proteins.

A greater than 2-fold increase in mitotic index and significant increase in the —SH level of the cytoplasmic soluble proteins at 12 hours is also found as a result of 20-methylcholanthrene administration (Table II, columns 6 and 4). A reducing cellular milieu is known to coincide with high anabolic rate which, in turn, is dependent on the high rate of synthesis of nucleic acids. The increase of soluble sulfhydryl compounds in the cytoplasm may be required to maintain, in the reduced —SH state, enzymes which support pathways of nucleic acid synthesis and, thus, to shift metabolic equilibrium toward anabolism. However, such increase of nucleic acid synthesis, induced by 20-methylcholanthrene, would apparently be mediated through different route(s) than that following partial hepatectomy, since no decrease in the proposed growth regulatory agent (*h* protein) is found in the former case.

*Administration of diethylnitrosamine*

In the studies of Sorof, Young and Ott (1958) on the liver *h* protein level of rats following administration of carcinogenic azo dyes and 2-acetylaminofluorene, determinations were made at those time intervals where previous studies had yielded maximum liver protein-bound dyes and, for 2-acetylaminofluorene, where the livers of rats showed similar histological changes. In the present study determinations were made at 7 intervals up to 10 weeks of diethylnitrosamine administration. As with the carcinogenic azo dyes and 2-acetylaminofluorene at the time intervals studied by Sorof, Young and Ott (1958), an increase in the amount of *h* protein was found during the entire period of diethylnitrosamine administration; the most dramatic increase was observed at 1 week (Table III, column 2).

The increase in *h* protein level resulting from the feeding of diethylnitrosamine was paralleled by a considerable increase in the amount of isolable soluble liver proteins (Table III, column 3). This finding was unexpected since diethylnitrosamine and its methyl homolog, dimethylnitrosamine, are known to be potent inhibitors of protein synthesis both *in vitro* and *in vivo* as measured by the incorporation of radioactively labelled amino acids (Magee, 1958; Brouwers and Emmelot, 1960; Hultin, Arrhenius, Löw and Magee, 1960). Two explanations may be offered. One is that the inhibition of protein synthesis described by the above authors was observed only a few hours after injection of or treatment with diethylnitrosamine or dimethylnitrosamine. It is thus not impossible that the considerable rise of total soluble proteins at 1 week is due to an increase of protein synthesis taking place after a temporary initial inhibition. A second possibility is suggested by the fact that dimethylnitrosamine is known to cause rapid early disintegration of the membrane of the endoplasmic reticulum and dispersion of the ribosomes throughout the cytoplasm (Emmelot and Benedetti, 1960); the lipoprotein remnants of the disintegrated membranes could thus remain in the supernatant during sedimentation, contributing to the protein content of the lyophilizate.

There is considerable evidence that sulfhydryl groups play crucial roles in the carcinogenic process, and this has been exhaustively reviewed by Harington (1967). Although the manner of involvement of the —SH groups in carcinogenesis is still a subject of hypothesis, hepatocarcinogenic substances, in general, bring about an increase in the —SH level in the liver. For example, a substantial increase of reduced glutathione in the liver was found at all weekly intervals up to 8 weeks in rats receiving 0·25% DL-ethionine in the diet (Hsu and Geller, 1967); also, feeding a single dose of the azo carcinogens, 3′-methyl-4-dimethylaminoazobenzene or 4-dimethylaminoazobenzene, to rats causes a significant increase in trichloroacetic acid-soluble —SH groups in the liver after 40 hours (Dijkstra, 1964). Thus, the significant increase in the sulfhydryl level of soluble rat liver proteins (Table III, column 4), found as the result of diethylnitrosamine feeding over a 10 week period, is as would be expected for this hepatocarcinogen. The concomitant increase in mitotic index (Table III, column 5) concurs with the suggestion of Együd and Szent-Györgyi (1966) that —SH groups act as a common denominator for many of the single processes involved in cell division.

### SUMMARY

1. Following partial hepatectomy the electrophoretic total *h* protein fraction of the rat liver soluble proteins shows a large, reversible decrease with a minimum at 37 hours. The decrease of the *h* protein level coincides with a reversible rise of sulfhydryl level in the total soluble proteins and with a reversible increase in mitotic rate in the liver tissue.

2. Intraperitoneal injection of 20-methylcholanthrene to weanling rats does not bring about change in the *h* protein level. There is, however, a considerable temporary rise of the mitotic rate reaching a maximum at 24 hours, and this is accompanied by an increase of the sulfhydryl level of the total soluble proteins of the liver.

3. During an entire 10 week period of continuous administration of diethylnitrosamine to rats elevated *h* protein levels are observed, the highest occurring at 1 week. There is a rough parallelism between *h* protein level and sulfhydryl content of the total soluble liver proteins. The mitotic rate of the liver tissue gradually increases during the period of carcinogen administration. Diethylnitrosamine-induced liver tumors showed *h* protein levels well below those of normal liver.

These investigations were supported by United States Public Health Service Research Grants CA-05431 and CA-05793 from the National Cancer Institute. Mitotic indexes of the partial hepatectomy experiments were read by Dr. Clinton A. Olmstead, Delta Research Institute consultant service. The expert technical assistance of Mrs. Shirley B. Bemis and Miss Mattie J. Tison is gratefully acknowledged.

### REFERENCES

Arcos, J. C. and Arcos, M.—(1958) *Biochim. biophys. Acta*, **28**, 9.
Arcos, J. C., Conney, A. H. and Buu-Hoi, Ng. Ph.—(1961) *J. biol. Chem.*, **236**, 1291.
Arcos, J. C., Gosch, H. H. and Zickafoose, D.—(1961) *J. biophys. biochem. Cytol.*, **10**, 23.
Argus, M. F., Arcos, J. C., Mathison, J. H. and Alam, A.—(1966) *Arzneimittel-Forsch.*, **16**, 1083.
Bakay, B. and Sorof, S.—(1964) *Cancer Res.*, **24**, 1814.
Becker, F. F. and Broome, J. D.—(1967) *Science, N.Y.*, **156**, 1602.
Brouwers, J. A. J. and Emmelot, P.—(1960) *Expl Cell Res.*, **19**, 467.
Calva, E. and Cohen, P. P.—(1959) *Cancer Res.*, **19**, 679.
Canellakis, E. S.—(1957) *J. biol. Chem.*, **227**, 329.
Canellakis, E. S., Jaffe, J. J., Mantsavinos, R. and Krakow, J. S.—(1959) *J. biol. Chem.*, **234**, 2096.
Chinard, F. P. and Hellerman, L.—(1954) ‘ Determination of Sulfhydryl Groups in Certain Biological Substances.’ *In* ‘ Methods of Biochemical Analysis ’ edited by Glick, D. New York (Interscience). Vol. I, pp. 1–26.
Dijkstra, J.—(1964) *Br. J. Cancer*, **18**, 608.
Együd, L. G. and Szent-Györgyi, A.—(1966) *Proc. natn. Acad. Sci. U.S.A.*, **52**, 388.
Emmelot, P. and Benedetti, E. L.—(1960) *J. biophys. biochem. Cytol.*, **7**, 393.
Fraser, L. B. and Cater, D. B.—(1967) *Br. J. Cancer*, **21**, 235.
Freed, J. J. and Sorof, S.—(1966) *Biochem. biophys. Res. Commun.*, **22**, 1.
Hämmerling, J.—(1939) *Biol. Zbl.*, **59**, 158.—(1953) *Int. Rev. Cytol.*, **2**, 475.
Harington, J. S.—(1967) *Adv. Cancer Res.* **10**, 247.
Hecht, L. I. and Potter, V. R.—(1956) *Cancer Res.*, **16**, 988.
Hellerman, L., Chinard, F. P. and Dietz, V. R.—(1943) *J. biol. Chem.*, **147**, 443.
Hopsu, V. R. and Harkonen, M.—(1960) *Acta. path. microbiol. scand.*, **48**, 94.
Hsu, J. G. and Geller, S.—(1967) *Archs Biochem. Biophys.*, **118**, 85.
Hultin, T., Arrhenius, E., Löw, H. and Magee, P. N.—(1960) *Biochem. J.*, **76**, 109.
Lowry, O. H., Rosebrough, N. J., Farr, A. L. and Randall, R. J.—(1951) *J. biol. Chem.*, **193**, 265.
Magee, P. N.—(1958) *Biochem. J.*, **70**, 606.

Miller, E. C. and Miller, J. A.—(1947) *Cancer Res.*, 7, 468.
Miller, E. C., Miller, J. A., Sapp, R. W. and Weber, G. M.—(1949) *Cancer Res.*, **9**, 336.
Miller, J. A. and Miller, E. C.—(1953) *Adv. Cancer Res.*, **1**, 339.
Potter, V. R.—(1958) *Fedn Proc. Fedn Am. Socs exp. Biol.*, **17**, 691.
Setnikar, I. and Magistretti, M. J.—(1965) *Arzneimittel-Forsch.*, **15**, 1042.
Sorof, S., Claus, B. and Cohen, P. P.—(1951) *Cancer Res.*, **11**, 873.
Sorof, S. and Cohen, P. P.—(1951) *Cancer Res.*, **11**, 376.
Sorof, S., Young, E. M. and Fetterman, P. L.—(1960) *Expl Cell Res.*, **20**, 253.
Sorof, S., Young, E. M., Luongo, L., Kish, V. M. and Freed, J. J.—(1967) ' Inhibition of Cell Multiplication *in vitro* by Liver Arginase '. *In* ' Wistar Institute Symposium Monograph on Growth Regulating Substances for Animal Cells in Culture ', edited by Defendi, V. and Stoker, M. Philadelphia (The Wistar Institute Press), No. 7, pp. 25–38.
Sorof, S., Young, E. M., McBride, R. Z. and Coffey, C. B.—(1965) *Fedn Proc. Fedn Am. Socs exp. Biol.*, **24**, 685.
Sorof, S., Young, E. M., McCue, M. M. and Fetterman, P. L.—(1963) *Cancer Res.*, **23**, 864.
Sorof, S., Young, E. M. and Ott, M. G.—(1958) *Cancer Res.*, **18**, 33.
Wirtz, G. H., and Arcos, J. C.—(1958) *Experientia*, **14**, 177.


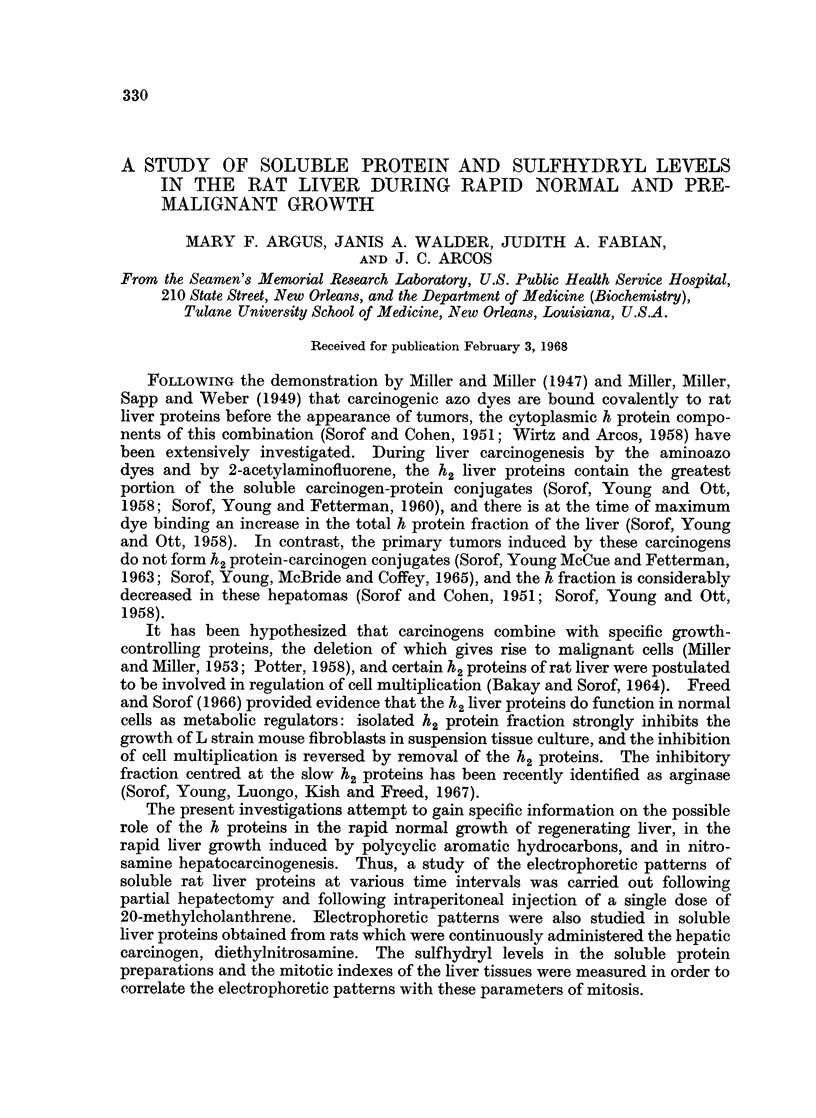

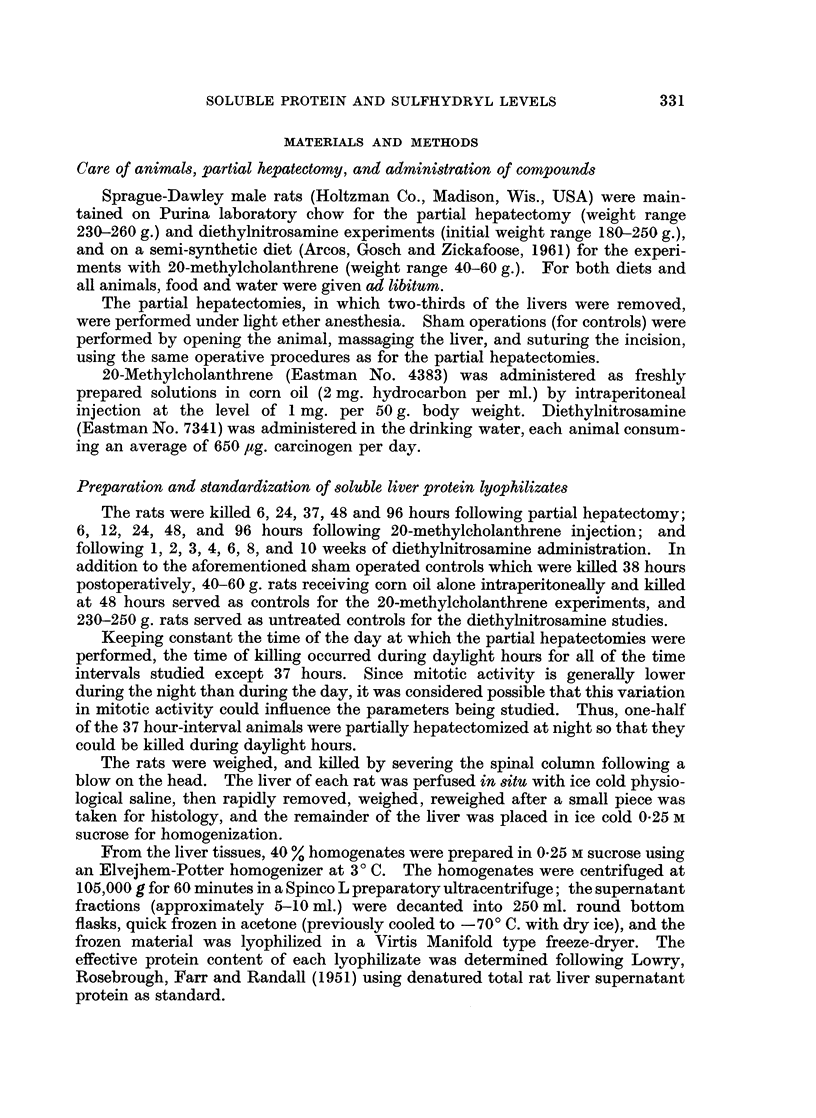

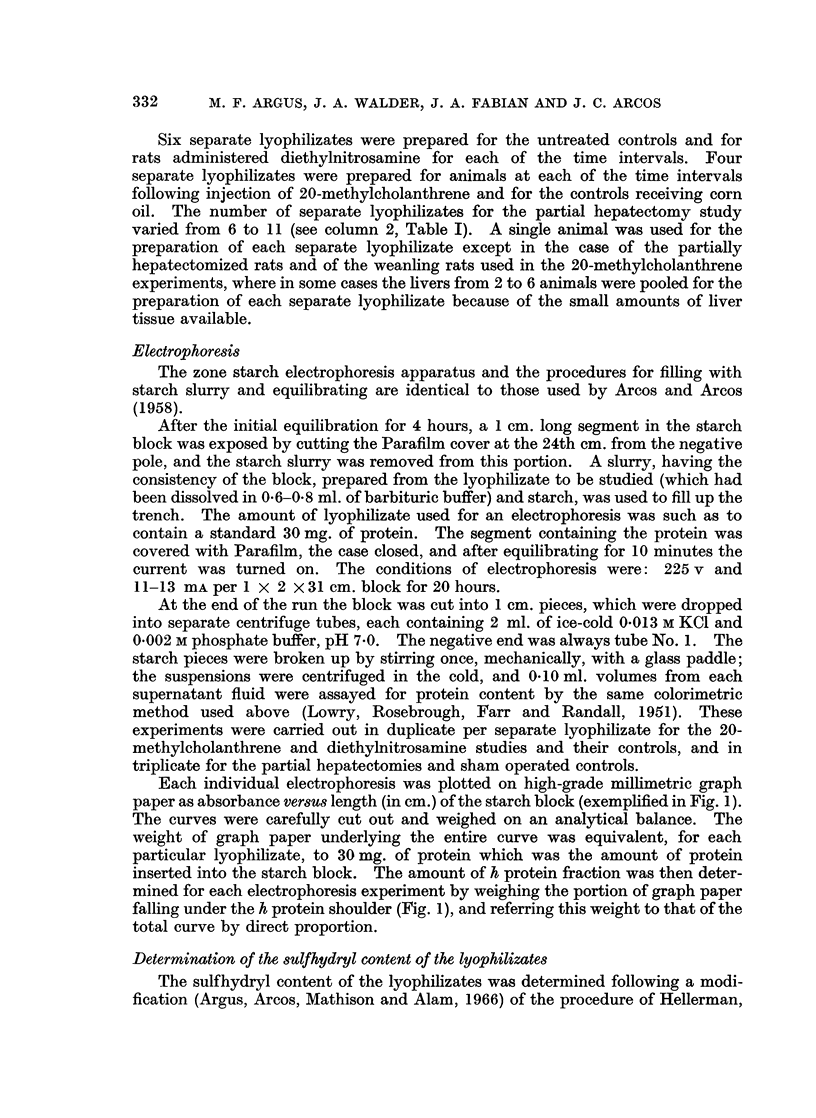

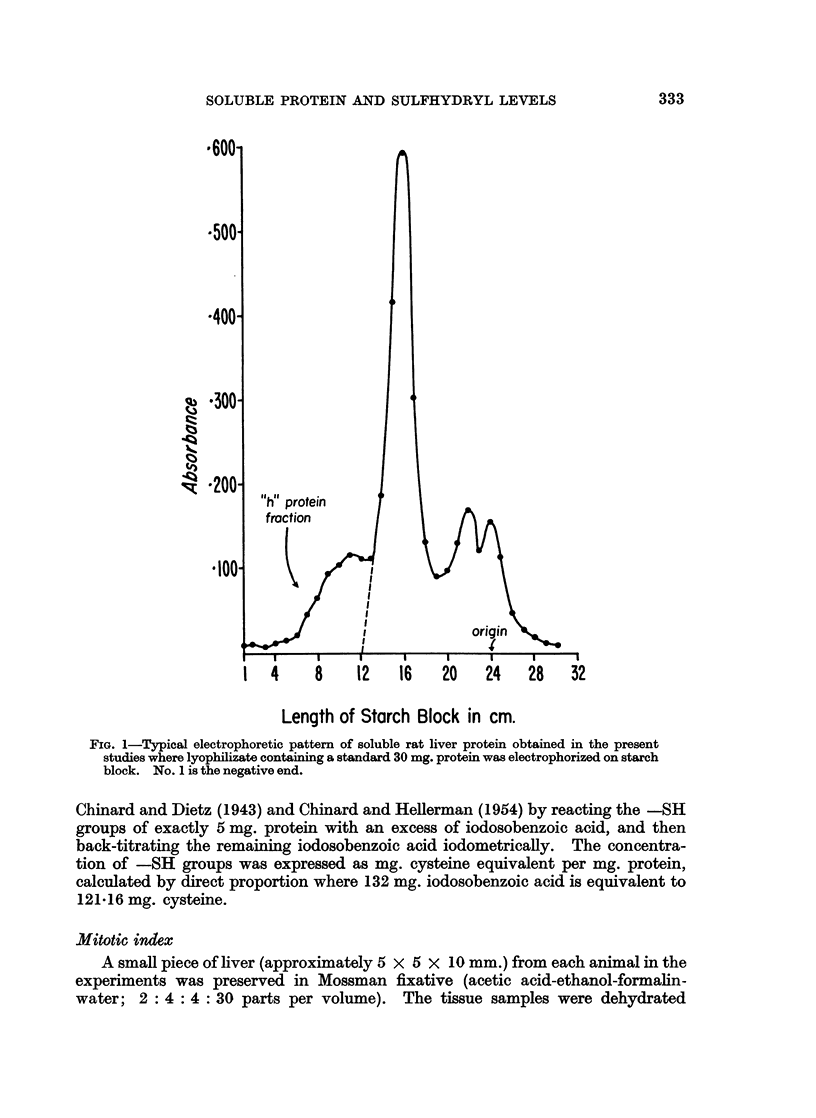

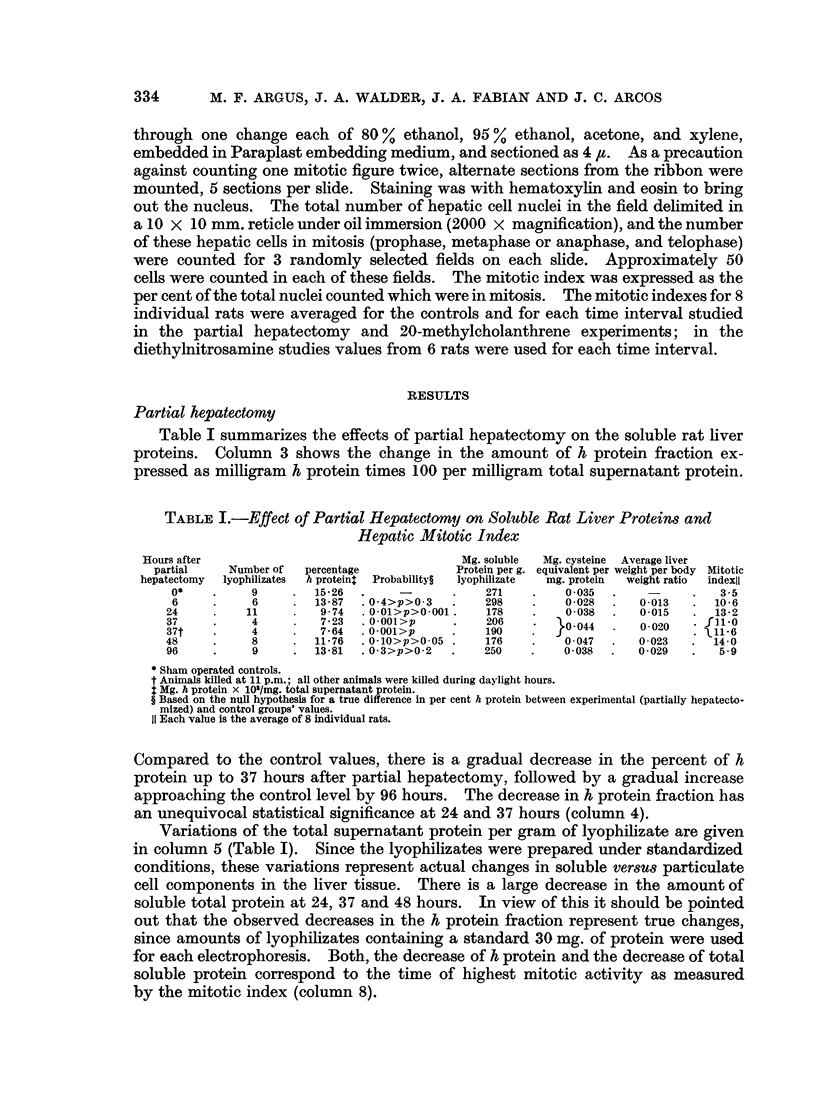

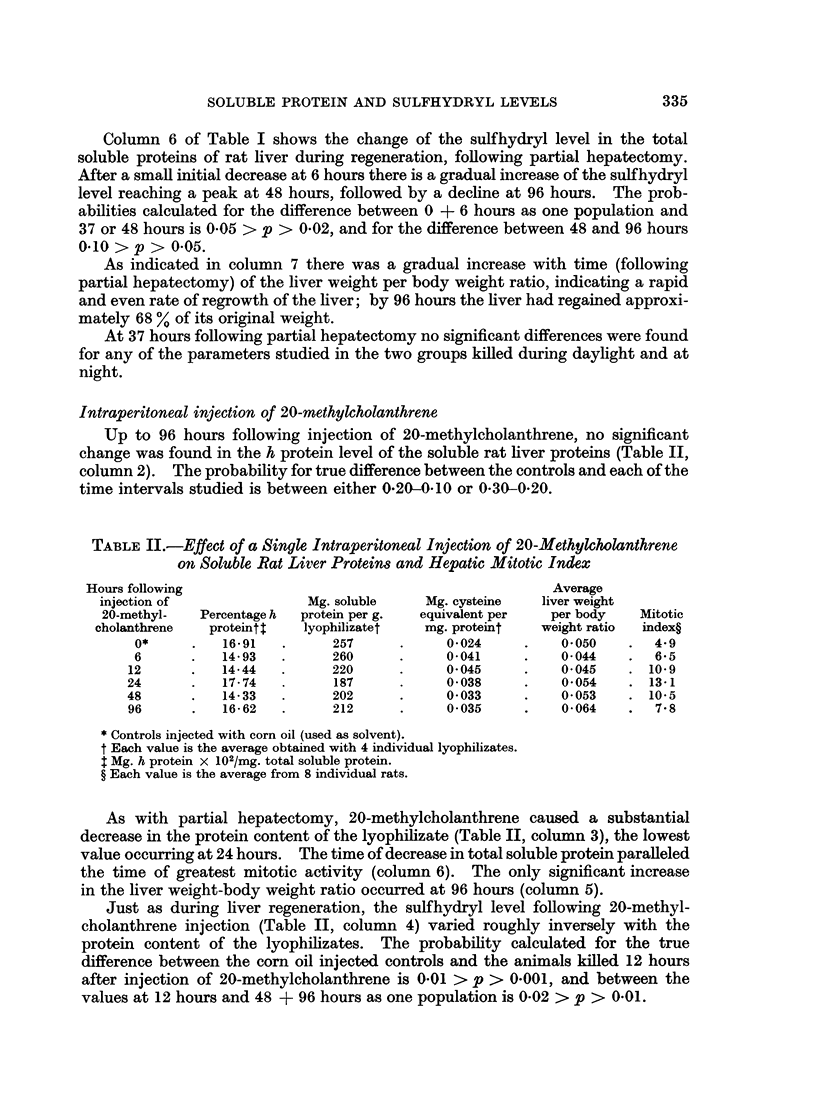

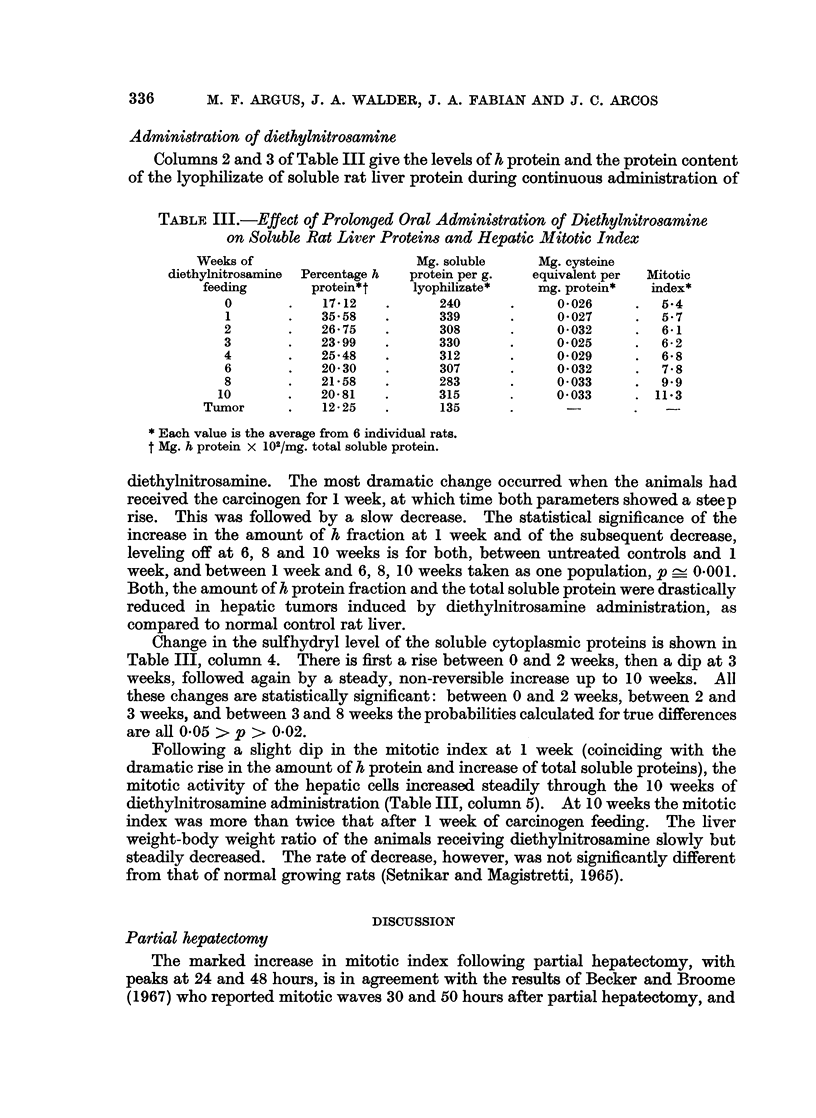

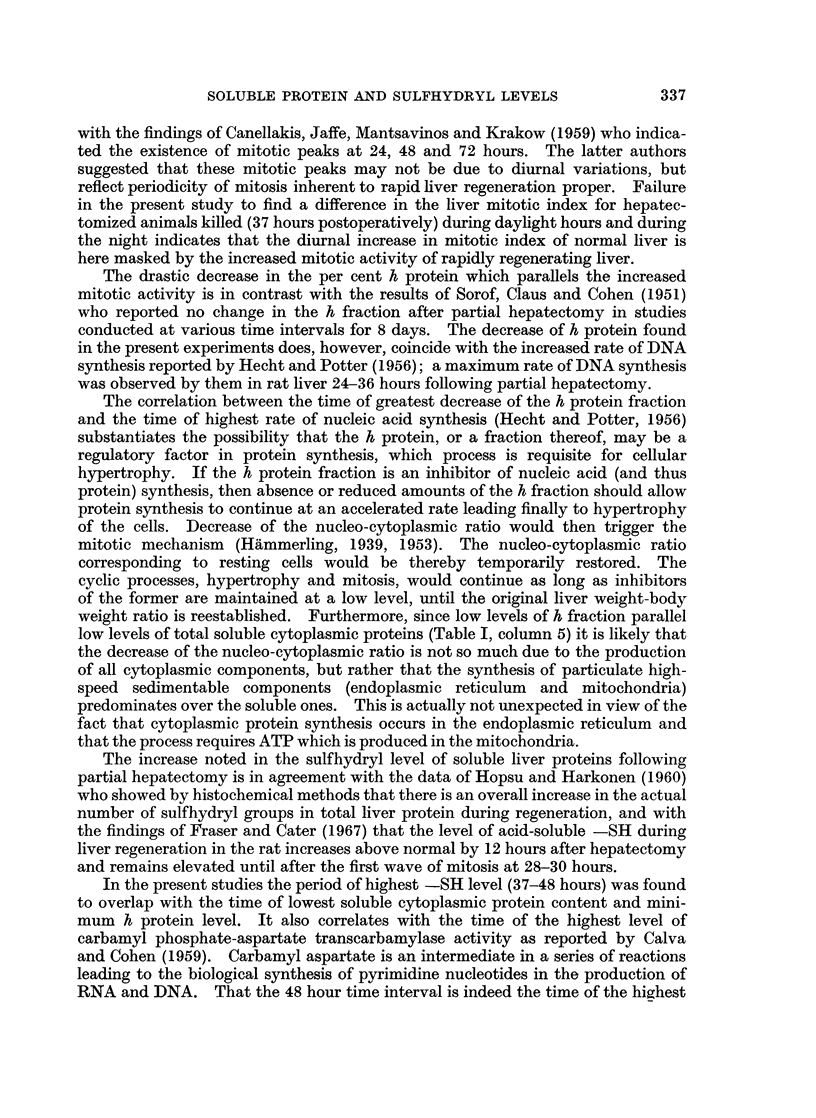

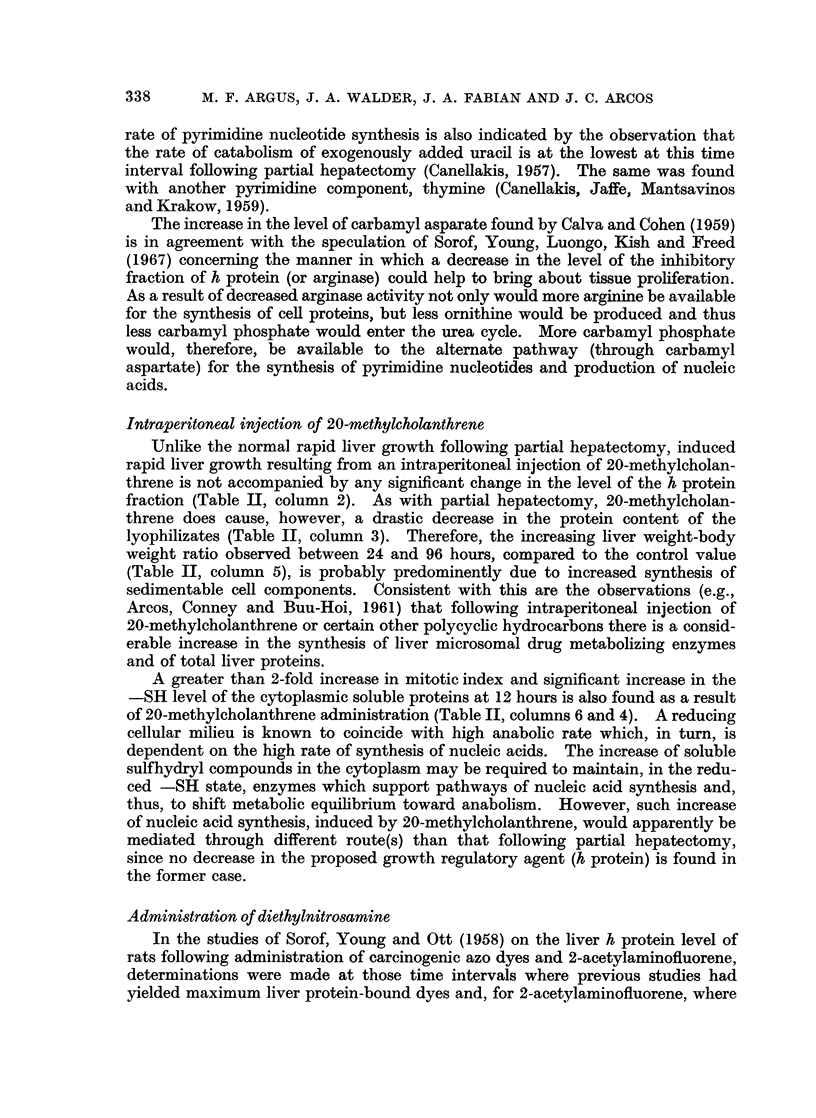

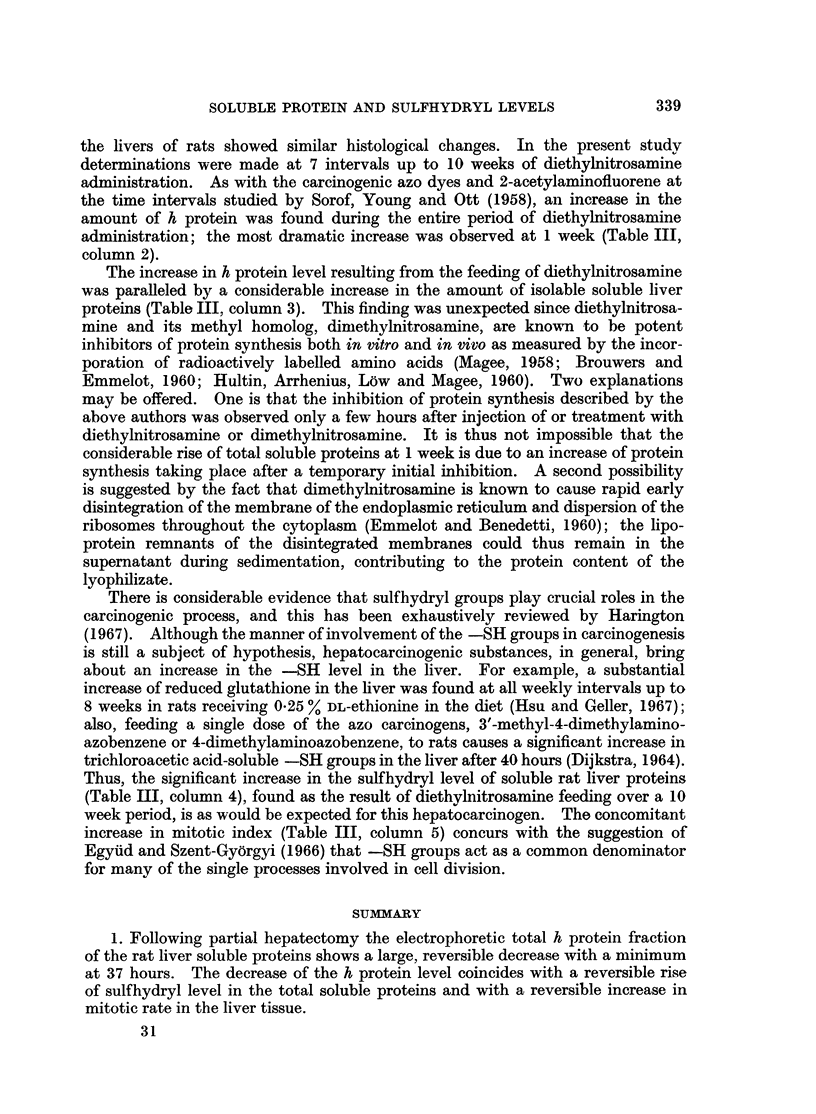

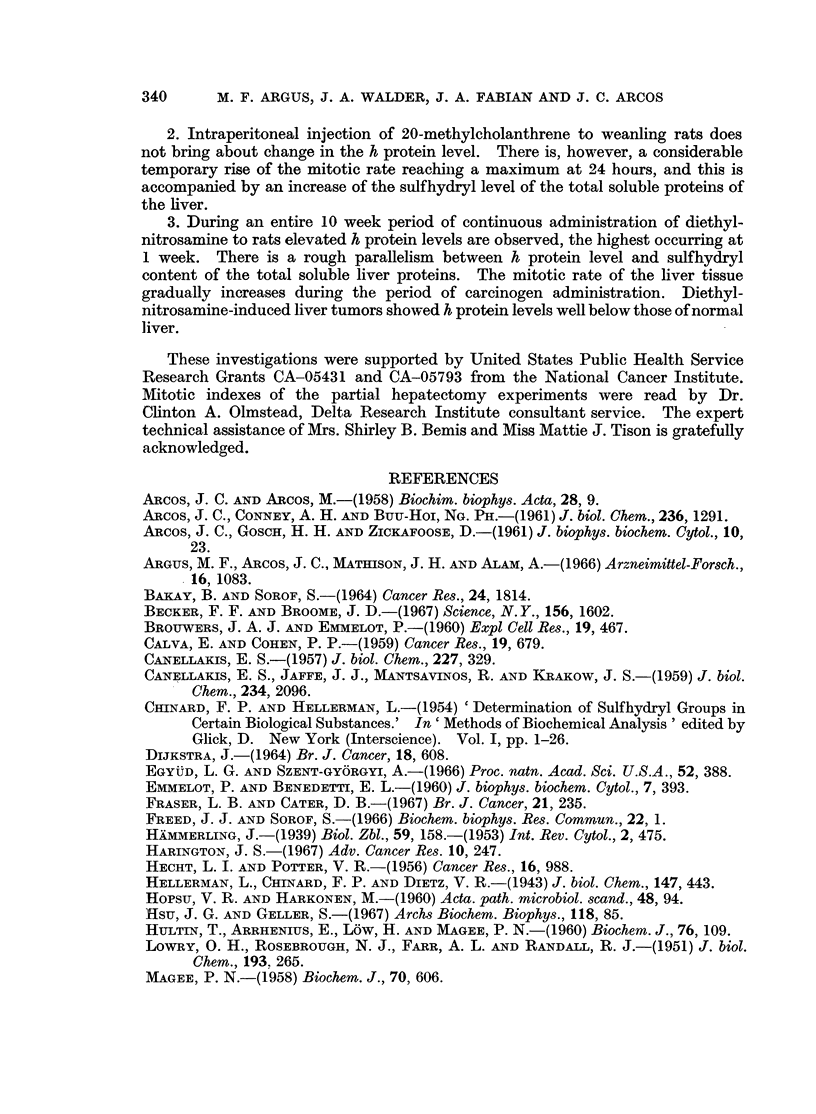

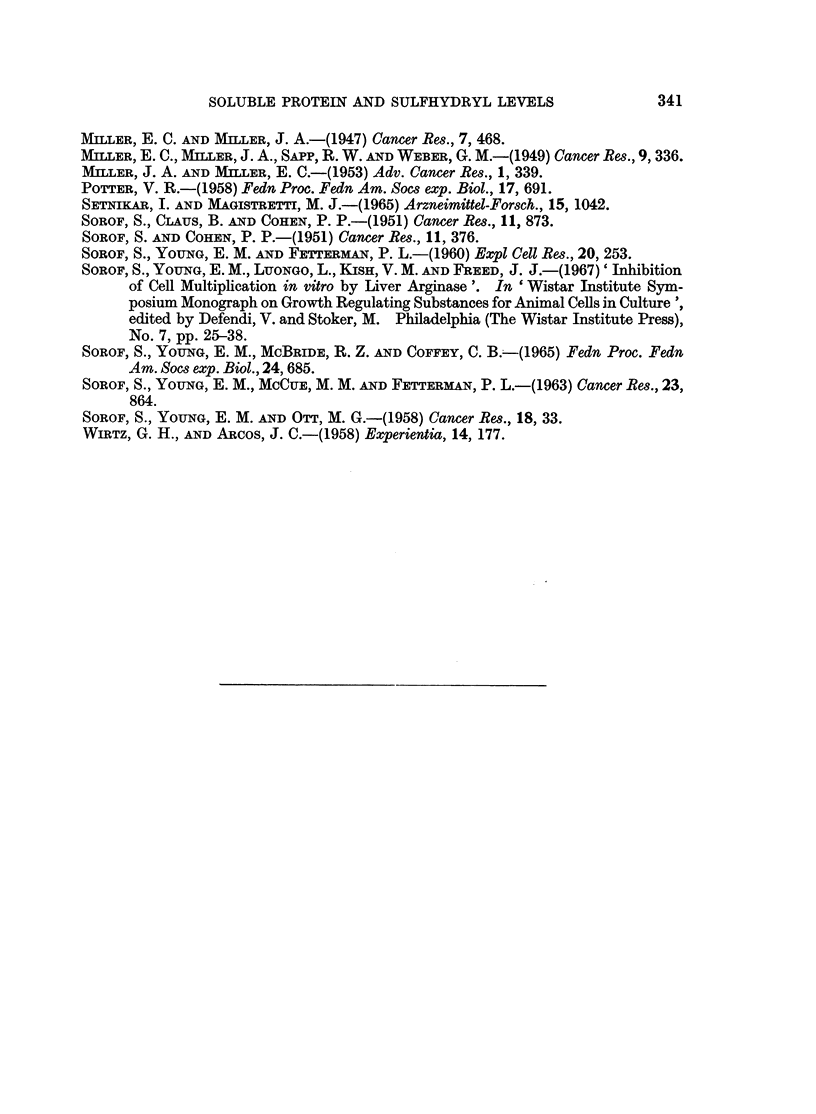


## References

[OCR_00605] ARCOS J. C., CONNEY A. H., BUU-HOI N. P. (1961). Induction of microsomal enzyme synthesis by polycyclic aromatic hydrocarbons of different molecular sizes.. J Biol Chem.

[OCR_00609] Argus M. F., Arcos J. C., Mathison J. H., Alam A. (1966). Studies on the denaturation of biological macromolecules by chemical carcinogens.. Arzneimittelforschung.

[OCR_00615] Becker F. F., Broome J. D. (1967). L-asparaginase: inhibition of early mitosis in regenerating rat liver.. Science.

[OCR_00623] CANELLAKIS E. S., JAFFE J. J., MANTSAVINOS R., KRAKOW J. S. (1959). Pyrimidine metabolism. IV. A comparison of normal and regenerating rat liver.. J Biol Chem.

[OCR_00619] CANELLAKIS E. S. (1957). Pyrimidine metabolism. II. Enzymatic pathways of uracil anabolism.. J Biol Chem.

[OCR_00625] CHINARD F. P., HELLERMAN L. (1954). Determination of sulfhydryl groups in certain biological substances.. Methods Biochem Anal.

[OCR_00629] DIJKSTRA J. (1964). THE CONTENTS OF TRICHLOROACETIC ACID-SOLUBLE SULPHYDRYL COMPOUNDS AND ASCORBIC ACID IN THE LIVER OF RATS FED AMINOAZO DYES: THE EFFECT OF A SINGLE LARGE DOSE OF DYE.. Br J Cancer.

[OCR_00631] Együd L. G., Szent-Györgyi A. (1966). Cell division, SH, ketoaldehydes, and cancer.. Proc Natl Acad Sci U S A.

[OCR_00633] Fraser L. B., Cater D. B. (1967). Variation of acid-soluble sulphydryl groups during liver regeneration.. Br J Cancer.

[OCR_00639] HECHT L. I., POTTER V. R. (1956). Nucleic acid metabolism in regenerating rat liver. I. The rate of deoxyribonucleic acid synthesis in vivo.. Cancer Res.

[OCR_00637] Harington J. S. (1967). The sulfhydryl group and carcinogenesis.. Adv Cancer Res.

[OCR_00649] LOWRY O. H., ROSEBROUGH N. J., FARR A. L., RANDALL R. J. (1951). Protein measurement with the Folin phenol reagent.. J Biol Chem.

[OCR_00653] MAGEE P. N. (1958). Toxic liver injury; inhibition of protein synthesis in rat liver by dimethylnitrosamine in vivo.. Biochem J.

[OCR_00663] SOROF S., CLAUS B., COHEN P. P. (1951). Effect of regeneration and inanition on the electrophoretic properties of soluble rat liver proteins.. Cancer Res.

[OCR_00668] SOROF S., YOUNG E. M., PETTERMAN P. L. (1960). Electrophoretic distribution of derivatives of 2-acetyl-aminofluorene-9-C14 bound to soluble proteins of rat liver in vivo.. Exp Cell Res.

[OCR_00684] WIRTZ G. H., ARCOS J. C. (1958). Studies on the dye-binding fraction of soluble liver proteins from rats fed aminoazo dyes.. Experientia.

